# Urinary Tetrabromobenzoic Acid (TBBA) as a Biomarker of Exposure to the Flame Retardant Mixture Firemaster^^®^^ 550

**DOI:** 10.1289/ehp.1308028

**Published:** 2014-05-13

**Authors:** Kate Hoffman, Mingliang Fang, Brian Horman, Heather B. Patisaul, Stavros Garantziotis, Linda S. Birnbaum, Heather M. Stapleton

**Affiliations:** 1 Nicholas School of the Environment, Duke University, Durham, North Carolina, USA; 2 Department of Biology, North Carolina State University, Raleigh, North Carolina, USA; 3 National Institute of Environmental Health Sciences, National Institutes of Health, Department of Health and Human Services, Research Triangle Park, North Carolina, USA; 4 National Cancer Institute, National Institutes of Health, Department of Health and Human Services, Research Triangle Park, North Carolina, USA; * These authors contributed equally to this work.

## Abstract

Background: Firemaster® 550 (FM550) is commonly added to residential furniture to reduce its flammability. Recent toxicological evidence suggests that FM550 may be endocrine disrupting and obesogenic.

Objectives: Our objectives were to develop methods to assess exposure to FM550 in human populations and to identify potential routes of exposure.

Methods: Using mass spectrometry methods, we developed a method to measure 2,3,4,5-tetrabromobenzoic acid (TBBA), a urinary metabolite of the major brominated FM550 component 2-ethylhexyl-2,3,4,5-tetrabromobenzoate (TBB). The method was applied to a cohort of adult volunteers (*n* = 64). Participants completed questionnaires, provided urine and handwipe samples, and collected dust samples from their homes. We measured TBB and the other major brominated FM550 component, bis(2-ethylhexyl)-2,3,4,5-tetrabromophthalate (TBPH), in paired dust and handwipe samples.

Results: TBBA was detected in 72.4% of urine samples. Although TBBA is a rapidly formed metabolite, analyses indicated moderate temporal reliability (interclass correlation coefficient = 0.56; 95% confidence interval: 0.46, 0.66). TBB and TBPH were detected frequently in dust samples [geometric mean (GM) = 315.1 and 364.7 ng/g, respectively] and in handwipes (GM = 31.4 and 23.4 ng, respectively). Levels of TBB and TBPH in dust were positively correlated with levels in handwipes. In addition, levels of TBB in handwipes were positively correlated with urinary TBBA. Results suggest frequent hand washing may reduce the mass of TBB on participants’ hands and reduce urinary TBBA levels.

Conclusions: Cumulatively, our data indicate that exposures to FM550 are widespread and that the home environment may be an important source of exposure. Urinary TBBA provides a potentially useful biomarker of FM550 exposure for epidemiologic studies.

Citation: Hoffman K, Fang M, Horman B, Patisaul HB, Garantziotis S, Birnbaum LS, Stapleton HM. 2014. Urinary tetrabromobenzoic acid (TBBA) as a biomarker of exposure to the flame retardant mixture Firemaster® 550. Environ Health Perspect 122:963–969; http://dx.doi.org/10.1289/ehp.1308028

## Introduction

Flame retardant (FR) chemicals are commonly applied to raw materials and consumer products to reduce their flammability and meet fire safety standards. Until recently, polybrominated diphenyl ethers (PBDEs) accounted for a large portion of FRs added to polymers and resins used in furniture, electronics, and building materials (de Wit 2002). However, concern over their persistence, bioaccumulation, and toxicity led to voluntary phaseouts and bans of several commercial PBDE mixtures from the marketplace in many countries, including the United States (Costa et al. 2008; U.S. Environmental Protection Agency 2009).

To maintain compliance with consumer product flammability standards, various PBDE replacements have been developed and introduced into commerce (Covaci et al. 2011; Stapleton et al. 2008a; van der Veen and de Boer 2012). In 2003, Chemtura introduced Firemaster® 550 (FM550) as a replacement for the commercial pentabrominated diphenyl ether (pentaBDE) mixture used in polyurethane foam applications (Stapleton et al. 2008a). Stapleton et al. (2008a) identified four main components of FM550: *a*) triphenyl phosphate (TPP); *b*) a mixture of isopropylated triphenyl phosphate isomers (ITPs); *c*) 2-ethylhexyl-2,3,4,5-tetrabromobenzoate (TBB); and *d*) bis(2-ethylhexyl)-2,3,4,5-tetrabromophthalate (TBPH). These components have been frequently detected in polyurethane foam samples from consumer goods, including furniture and baby products [e.g., nursing pillows and changing-table pads (Stapleton et al. 2009, 2011)]. Based on these two studies (Stapleton et al. 2009, 2011), FM550 appears to be the second most common FR mixture applied to foam-containing products. However, with the expected phaseout of tris(1,3-dichloroisopropyl)phosphate (TDCPP), use of FM550 may increase.

Like PBDEs, FM550 is an additive FR (i.e., not chemically bound to products) and its components leach into the environment over time, as evidenced by the prevalence of TBB and TBPH in indoor dust, outdoor air, and sewage (Ali et al. 2012; Covaci et al. 2011; Dodson et al. 2012; La Guardia et al. 2010; Ma et al. 2012; Stapleton et al. 2008a, 2009). These data suggest that the general population comes into contact with FM550 frequently and widespread exposure is likely; however, potential exposure sources and routes of human exposures have yet to be investigated. Based on similarities between FM550 and PBDEs (e.g., modes of application, presence of brominated aromatic backbones), similar exposure pathways could be expected. For PBDEs, indoor dust has been identified as an important contributor to overall body burden, and data suggest that exposure via hands (e.g., incidental ingestion through hand-to-mouth contact) may in part explain how PBDEs get into the body (Johnson et al. 2010; Stapleton et al. 2012; Watkins et al. 2011).

Relatively little is known about the toxicokinetics of FM550 components. Recent *in vitro* evidence, however, suggested that TBB is metabolized to 2,3,4,5-tetrabromobenzoic acid (TBBA) in human hepatic tissues, whereas TBPH appears to be recalcitrant to metabolism (Roberts et al. 2012). The toxicities of FM550 components, as well as those of their potential metabolites (e.g., TBBA), are largely unknown because few studies have investigated the impacts of exposure. However, Patisaul et al. (2013) recently reported that FM550 is an endocrine disruptor and an obesogen; they found that pregnant rats exposed to FM550 across gestation and lactation had altered thyroid function and that their female offspring entered puberty early. In addition, offspring were 30–60% heavier by weaning, an effect that persisted into adulthood.

To our knowledge, no assessments of FM550 exposure and associated health outcomes have been carried out in human populations. Such studies rely on accurate means of assessing exposure; however, there is no biomarker of exposure to FM550, and data on environmental contributions to the body burden remain sparse. Thus, our primary objective was to develop methods to assess exposure to FM550 in human populations. We focused on the brominated FM550 components, and TBB in particular, because its use is thought to be limited to FM550 and another flame retardant mixture, Firemaster® BZ-54, whereas TBPH and the organophosphates in FM550 (i.e., TPP and ITPs) are used in numerous other flame retardant and plasticizer applications (Covaci et al. 2011; van der Veen and de Boer 2012). To support this approach, we developed an assay to measure TBBA in urine samples. Furthermore, we exposed six adult rats to a one-time dose of FM550 and measured TBBA in their urine over a 24-hr period. Using the newly developed method, we measured TBBA in urine samples from 64 healthy North Carolina adults. In addition, we assessed temporal variability in TBBA measures to inform exposure assessments in future epidemiologic studies. Finally, building on our previous work investigating environmental pathways of exposure to FRs, we assessed relationships between TBB and TBPH in household dust and handwipe samples and evaluated associations of environmental TBB and urinary TBBA as first steps in identifying possible sources and potential routes of human exposure to FM550.

## Materials and Methods

*Study design*. Two groups of healthy adult volunteers from central North Carolina were recruited to the National Institute of Environmental Health Sciences (NIEHS) Clinical Research Unit (CRU). Eligible participants were at least 18 years of age and had never been diagnosed with a kidney problem (not including kidney stones). One group of participants (*n* = 53) completed demographic and behavioral questionnaires, provided handwipe and urine samples, and collected dust samples in their homes (February–March 2012). All samples were collected from each participant within approximately 1 week. Another group of adults was recruited to provide repeated urine samples on 5 consecutive days (11 participants with 53 total urine samples, all collected in August 2011). All study protocols were approved by the NIEHS Institutional Review Board, and participants provided informed consent.

*Questionnaires*. We designed the questionnaire to collect information on personal characteristics, including age, sex, race and ethnicity, and height and weight, as well as information on personal habits, such as the average number of hours spent active in the home and the average number of times participants washed their hands each day. Information on hand washing was collected as never, 1–2 times/day, 3–5 times/day, 6–8 times/day, and > 8 times/day. For analyses, we collapsed hand washing into two categories: ≤ 8 times/day (low) and > 8 times/day (frequent), with the categorization determined based on the distribution of responses. Participants also provided information about whether they used hand-sanitizer gels (yes or no). Response categories for average time spent active in the home and average time spent driving each day were also dichotomized to ≤ 8 hr/day and > 8 hr/day, and ≤ 1 hr/day and > 1 hr/day, respectively.

*Dust collection*. Each participant was provided with a kit and instructions for the collection of house dust samples (see Supplemental Material, Figure S3). Participants inserted a dust collection unit, which contains a nylon thimble, into the hose attachment of their home vacuum cleaners and followed instructions to vacuum the floor in the main living area of their homes for 2 min. The dust collection unit, containing the thimble, was them removed from the vacuum, sealed in a plastic bag, and mailed back to the CRU. The nylon thimbles were never in contact with the plastic bag. Upon receipt in the laboratory, the thimbles were removed, and the dust sieved to < 500 μm and stored in amber glass vials at room temperature until analysis.

*Handwipe collection*. Handwipe samples were collected at the CRU using previously described methods (Stapleton et al. 2008b). Briefly, clinical staff wiped the entire surface of each participant’s hands with a sterile gauze wipe soaked in 3.0 mL isopropyl alcohol, wiping each hand two times from the fingers to the wrist, including the space between fingers and both sides. Wipes were sealed in individual plastic bags and stored at –20°C until analysis.

*Dust and handwipe analyses*. Handwipe and dust samples were extracted in the laboratory and analyzed for TBB and TBPH using minor modifications of previously published methods (Stapleton et al. 2014; Van den Eede et al. 2012). Detailed information on the methods and standards are provided in Supplemental Material (“Dust and handwipe analyses,” p. 2). Handwipes and dust samples (~ 100 mg) were spiked with internal standards and extracted with 50:50 dichloromethane:hexane using either Soxhlets or sonication, respectively. Extracts were concentrated to 1.0 mL and further cleaned using a solid-phase extraction cartridge (Supelclean ENVI-Florisil, 6 mL, 500 mg bed weight; Supelco). TBB and TBPH were eluted with hexane, reduced in concentration to 1.0 mL, spiked with recovery standards, and analyzed using gas chromatography mass spectrometry operated in electron capture negative ionization mode. Analysis of laboratory blanks (*n* = 5) and an indoor dust standard reference material (SRM 2585; NIST) were used for quality assurance and quality control.

*Urinary elimination in rats*. To further investigate metabolism of TBB *in vivo*, we conducted an assessment of TBB metabolism in Wistar rats (mean weight, 281 g; range, 257–336 g; mean age, 4.3 months; age range, 4.0–4.4 months). An ethanol solution of FM550 was prepared from a commercial stock in the same manner as described previously (Patisaul et al. 2013) to obtain a final concentration of 0.05 mg/μL. For these experiments, we used adult female Wistar rats (*n* = 6/treatment), obtained from the same in-house colony as for our previous study to ensure consistency with that work (Patisaul et al. 2013). The animals used for the present study can develop an obese phenotype (Patisaul et al. 2013). The sample size used is consistent with, or higher than, what is typically used to examine the pharmacokinetics of endocrine-disrupting chemicals (EDCs), such as bisphenol A (BPA), in rodents (Churchwell et al. 2014; Taylor et al. 2011). Animals were maintained on a soy-free diet (Teklad 2020; Harlan Laboratories) in an environment that minimizes exogenous EDC exposure (including the use of glass water bottles, wood chip bedding, polysulfone caging, and filtered water) at the North Carolina State University (NCSU) Biological Resources Facility (Patisaul et al. 2012, 2013). Study animals (pair-housed until day of exposure and testing) were kept in a climate-controlled room at 25°C and 45–60% average relative humidity and a 12-hr light cycle (lights on from 1200 hours to 2400 hours). At 1000 hours, rats (*n* = 6) were exposed to FM550 by dispensing 20 μL of FM 550 solution (1 mg) onto one-fourth of a soy-free food treat pellet (apple- or chocolate-flavored AIN-76A Rodent Diet Test Tabs; Test Diet). Control rats (*n* = 6) were exposed to the treat pellet spiked with 20 μL of ethanol alone. Over the 24 hr following exposure, urine was collected from all 12 rats (at 0, 1 3, 6, and 24 hr). For each collection, we stimulated rats to urinate by moving them from their home cage to an unfamiliar clean cage with no bedding. All urinated within 3 min of being moved, and the urine was collected via pipette and stored at –20°C until analysis. Animal care and maintenance were conducted in accordance with the applicable portions of the Animal Welfare Act (2012) and the *Guide for the Care and use of Laboratory Animals* (National Research Council 2011) and were approved by the NCSU Institutional Animal Care and Use Committee. All procedures were approved and monitored by the supervising veterinarian for the duration of the project to ensure that all animals were treated humanely and with regard for the alleviation of suffering.

*Human urine collection and analysis*. Study participants provided spot urine samples during visits to the CRU (all samples were collected between 0830 and 1630 hours). Samples were collected into standard polypropylene specimen containers and were stored at –20°C until analysis. 2,3,5-Triiodobenzoic acid (TIBA; Sigma Aldrich) was used as the internal standard for quantification. The extraction and cleanup of TBBA in rat urine (< 1 mL for most samples) were performed based on the method used in our previous study (Roberts et al. 2012, in which 6 mL Agilent SampliQ OPT SPE columns (Agilent Technologies) were used to concentrate and clean the urine samples. However, we observed a matrix effect (i.e., ion suppression of TBBA and TIBA) in human urine, possibly due to the large volume of urine (~ 10 mL) extracted. Therefore, we modified the method to reduce matrix effects; these modifications are described in Supplemental Material (“Method development of TBBA analysis in human urine,” pp. 3–4, and Supplemental Material, Figure S1), along with more details on the quantification method. Briefly, after urine was thawed at room temperature, 10 mL was transferred to a precleaned 50 mL glass centrifuge tube and 5 ng TIBA was spiked in as an internal standard. The urine was further diluted with 10 mL phosphate buffer (pH 7.4; 0.1 M), and 1 mL concentrated sulfuric acid (95–98%; J.T. Baker) was added. After the diluted urine reached room temperature, 10 mL hexane was added to extract TBBA from the urine. The mixture was vortexed for 1 min and centrifuged at 3,000 rpm for 5 min. The supernant hexane layer was transferred into another empty 50 mL glass centrifuge tube, and the extraction step was repeated twice; all hexane was combined. For complete removal of sulfuric acid residue and further cleaning of the matrix, the extract was then washed twice with 10 mL deionized water that was acidified with 1 M acetic acid to pH 2–3. The final extract in hexane was blown down to approximately 1 mL using an automated nitrogen evaporation system. Extracts were then dried and reconstituted with 0.5 mL methanol:H_2_O (1:1, vol:vol) in liquid chromatography (LC) vials and analyzed using LC tandem mass spectrometry (LC/MS-MS) (Agilent 6410 Triple Quad LCMS) based on a previously described method (Roberts et al. 2012). In brief, TBBA and TIBA were analyzed in negative electrospray ionization mode using multiple reaction monitoring. We used ion transition *m/z* 436.6 to 392.6 for quantification of TBBA and *m/z* 436.6 to 79 for the qualifier ions. We used ion transition *m/z* 498.7 to 127 for quantification of TIBA and *m/z* 498.7 to 454.8 for the qualifier ions. Forty microliters of the extract was injected and separated by a Synergi Polar-RP column (50 × 2.0 mm, 2.5 μm; Phenomenex) with water and acetonitrile mobile phases with 5 mm acetic acid. In a matrix spike recovery evaluation, the average recovery of TBBA was 79 ± 9% in 10 urine samples. Because of the lack of a proper recovery standard, no additional chemical was used to calculate the recovery of TBBA in each urine sample. As part of our quality control, we also tested the interday and intraday variability of one TBBA detected urine sample, with a result of < 10%.

We also measured specific gravity (SG) in each urine sample using a digital handheld refractometer (Atago). SG measurements were used to produce TBBA concentrations corrected for urine dilution (Boeniger et al. 1993). Analyses were then conducted using both SG-corrected and raw measurements. Because the results were very similar with both methods, we present only the SG-corrected analyses. Creatinine, an alternative means of adjusting for dilution, was not measured in samples because it varies considerably by age and sex (James et al. 1988).

*Data analyses*. Quality control. We blank-subtracted the handwipe and house dust measurements using the average field (handwipe) or laboratory (dust) blank measurement. The method detection limits (MDLs) were calculated using three times the SD of the appropriate blanks. MDLs for TBB and TBPH measured in handwipes were 3.8 and 1.1 ng, respectively. MDLs for TBB and TBPH in house dust were 8.9 and 33.5 ng/g, respectively. Recovery of the internal standard, F-BDE-69, averaged 91 ± 18% in the handwipe samples and 88 ± 6% in the house dust samples. Measurements of TBB and TBPH in SRM 2585 averaged 35.2 ± 4.0 and 545 ± 74.5 ng/g, respectively, which are very similar to measurements made by Ali et al. (2012).

Statistical analyses. Concentrations below the MDL were substituted with the MDL divided by the square root of 2. Preliminary analyses indicated that concentrations of TBB and TBPH in handwipes and dust and concentrations of TBBA in urine were log-normally distributed. We calculated Pearson correlation coefficients (*r*_p_) using log_10_-transformed concentrations assess associations between continuous (log_10_-transformed) measures of TBB and TBPH in handwipes and dust, and TBBA in urine. We also calculated Spearman correlation coefficients (*r*_s_) using nontransformed values.

We used linear regression models to assess bivariate associations between demographic, behavioral, and environmental variables and TBB and TBPH in handwipes and TBBA in urine samples (outcomes were log_10_-transformed). To aid in the interpretation of results, we exponentiated beta coefficients (10^β^), producing the multiplicative change in outcome. As predictors of TBB and TBPH in handwipes, dust concentrations were categorized (dichotomized at the median and categorized into tertiles), and as predictors of urinary TBBA, both dust and handwipe concentrations were categorized to minimize the effect of skewed data and outliers in regression analyses.

We assessed temporal variability in TBBA concentrations by calculating interclass correlation coefficients (ICCs) (Shrout and Fleiss 1979). ICCs, which are calculated by dividing the between-subject variability by the sum of the between- and within-subject variability, provide a measure of the reliability of repeated measures over time (Rosner 2000). ICC values range from 0, indicating no reproducibility, to 1, indicating perfect reproducibility. All statistical analyses were performed in SAS (version 9.2; SAS Institute Inc.), with statistical significance defined as α ≤ 0.05 for main effects and α ≤ 0.20 for interactions.

## Results

**Figure 1:**
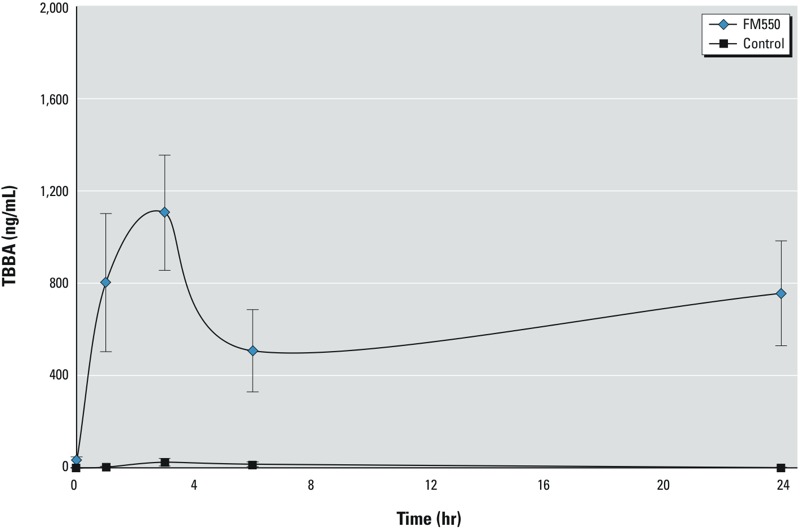
Mean (± SE) TBBA in urine samples from adult female rats administered either 1 mg FM550 or ethanol vehicle (control) one time via a treat pellet; *n = *6/treatment group. TBBA concentrations were measured at 1, 3, 6, and 24 hr after treatment (time zero).

*TBB metabolism*. TBBA was detected in urine from FM550-exposed rats and significantly increased during the first 2–3 hr after exposure, reaching a maximum concentration of 1,105 ng/mL. This was followed by a rapid decrease in concentration, which appeared to level off around 6–8 hr postexposure (Figure 1; see also Supplemental Material, Figure S2). These data indicate that urinary TBBA was rapidly formed from metabolism of FM550.

**Figure 2:**
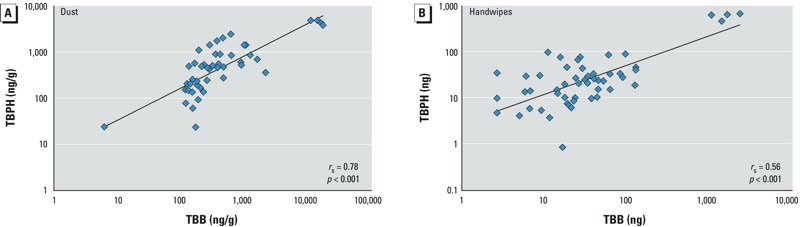
Correlation (*r*_s_) between TBB and TBPH in 49 dust samples (*A*) and 53 handwipe samples (*B*).

*FM550 in environmental samples*. The mean age of study participants was 44 years (range, 19–67 years). Approximately one-half were male (49.1%), and most reported white race (75.5%) and non-Hispanic ethnicity (94.3%). Table 1 presents summary statistics for TBB and TBPH concentrations in house dust and handwipes provided by the participants. Both compounds were detected in nearly all house dust samples (*n* = 93.9%). Geometric mean (GM) concentrations were 315.1 and 364.7 ng/g for TBB and TBPH, respectively (Table 1), with considerable variability among samples. TBB and TBPH levels in dust were highly correlated (*r*_p_ = 0.81, *p* < 0.001; *r*_s_ = 0.78, *p* < 0.001; Figure 2A; analysis robust to the exclusion of extreme observations). TBB and TBPH were also detected with high frequency in handwipe samples (92.5% and 98.1% of all samples), with GMs of 31.4 and 23.4 ng, respectively. As with dust, TBB and TBPH levels in handwipes were correlated, although the magnitude of the correlation was lower (*r*_p_ = 0.73, *p* < 0.001; *r*_s_ = 0.56, *p* < 0.001; Figure 2B).

**Table 1:** GMs and selected percentiles of TBB and TBPH in household dust and handwipes and of TBBA in urine samples from North Carolina adults contributing paired samples (*n* = 53).

Matrix and compound	*n *	Percent detects	GM	Perentile			Maximum
				25th	50th	75th	
Dust^*a*^
TBB (ng/g)	49	93.9	315.1	159.1	275.5	487.0	18148.7
TBPH (ng/g)	49	93.9	364.7	165.7	473.8	860.6	4814.2
Handwipes
TBB (ng)	53	92.5	31.4	14.8	26.9	55.5	2633.1
TBPH (ng)	53	98.1	23.4	9.9	23.0	40.8	655.1
SG-corrected urine^*a*^
TBBA (pg/mL)	52	76.9	5.6	2.3	5.3	10.8	340.6
^***a***^Insufficient sample volume for 4 dust samples and 1 urine sample.							

*Dust and handwipe associations*. In correlation analyses, continuous measures of TBB in handwipes and dust were positively correlated (*r*_p_ = 0.48; *p* < 0.001; *r*_s_ = 0.34; *p* = 0.01), as were levels of TBPH (r*_p_* = 0.38; *p* < 0.001; *r*_s_ = 0.35; *p* = 0.01). We used linear regression models to further explore bivariate associations between dust and handwipe concentrations. Table 2 presents exponentiated β-coefficients for these models, representing the multiplicative change in handwipe concentrations. For example, participants with high dust levels (tertile 3) in their homes had an average of 3.64 times the levels of TBB in their handwipe samples compared with participants with low TBB dust levels (tertile 1) (Table 2). Similarly, higher levels of TBPH in dust also contributed to higher mass of TBPH on participants’ hands (10^β^ = 2.81 for the tertile 3 compared with the tertile 1; 95% CI: 1.09, 7.22). For both TBB and TBPH, results were similar when we considered dichotomized versions of the dust (results not shown). Participants who reported frequent hand washing also tended to have decreased TBB mass on their hands (Table 2). Conversely, using hand-sanitizer gel had little effect on TBB levels in handwipes.

**Table 2:** Regression analyses for predictors of handwipe TBB and TBPH.

Predictor	TBB handwipes [10^β ^(95% CI)]	*p*-Value	TBPH handwipes [10^β ^(95% CI)]	*p*-Value
Sex
Male	Reference		Reference
Female	1.75 (0.75, 4.06)	0.19	1.62 (0.76, 3.55)	0.19
Age (years)	0.98 (0.94, 1.01)	0.18	0.99 (0.96, 1.02)	0.62
Average times hands washed/day
≤ 8 times/day	Reference		Reference
> 8 times/day	0.46 (0.18, 1.12)	0.08	0.81 (0.37, 1.78)	0.59
Hand sanitizer gel use
No	Reference		Reference
Yes	1.45 (0.58, 3.62)	0.41	1.62 (0.76, 3.55)	0.21
Average time active in the home/day
≤ 8 hr/day	Reference		Reference
> 8 hr/day	0.91 (0.34, 2.47)	0.85	1.51 (0.65, 3.55)	0.32
Average time driving in car/day
≤ 1 hr/day	Reference		Reference
> 1 hr/day	0.79 (0.34, 1.85)	0.58	0.95 (0.46, 1.95)	0.88
Dust TBB or TBPH
Low (tertile 1)	Reference		Reference
Mid (tertile 2)	1.09 (0.39, 3.06)	0.87	1.61 (0.64, 4.09)	0.31
High (tertile 3)	3.64 (1.27, 10.39)	0.02	2.81 (1.09, 7.22)	0.03
10^β^ Values represent the multiplicative change in handwipe levels relative to the reference group for categorical variables, or the per-unit change for continuous variables (age).				

**Figure 3:**
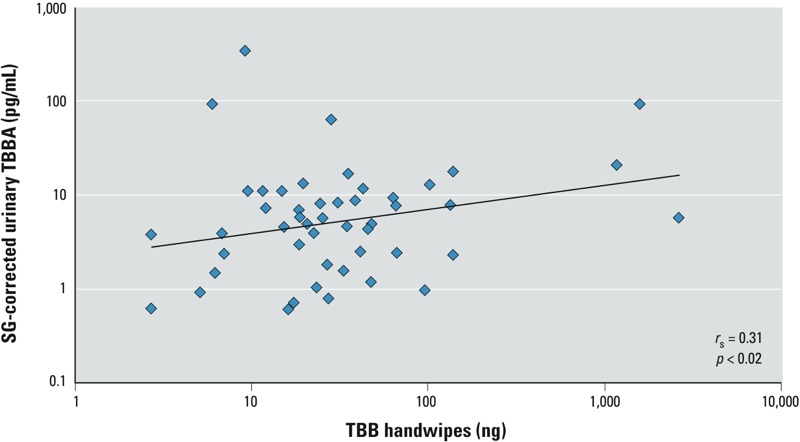
Correlation (*r*_s_) between TBB in handwipes and TBBA in urine samples.

*Handwipe and urine associations*. TBBA was detected frequently (76.9% detects) in urine samples from participants with paired house dust and handwipe samples, with a GM of 5.6 pg/mL (SG-corrected; Table 1). We observed a positive correlation between TBB levels in handwipes and TBBA in urine (*r*_p_ = 0.38; *p* < 0.001; *r*_s_ = 0.31; *p* = 0.02; Figure 3). Using linear regression models, we investigated bivariate associations between categorical handwipe TBB levels and TBBA (Table 3). Participants with high-TBB handwipe levels had 1.99 times the urinary TBBA concentrations of participants with low handwipe levels, although associations were imprecisely estimated (tertile 3 vs. tertile 1; 95% CI: 0.73, 5.41). In bivariate models, TBBA concentrations also tended to decrease with age (10^β^ = 0.97; 95% CI: 0.94, 1.00) and were lower for participants that spent > 1 hr/day in their cars (10^β^ = 0.54; 95% CI: 0.24, 1.21), although associations were imprecisely estimated.

**Table 3:** Regression analyses for predictors of urinary TBBA.

Predictor	SG-corrected TBBA [10^β^ (95% CI)]	*p*-Value
Sex
Male	Reference
Female	0.99 (0.44, 2.24)	0.98
Age (years)	0.97 (0.94, 1.00)	0.09
Average times hands washed/day
≤ 8 times/day	Reference
> 8 times/day	0.61 (0.25, 1.47)	0.27
Hand sanitizer gel use
No	Reference
Yes	0.88 (0.36, 2.14)	0.78
Average time active in the home/day
≤ 8 hr/day	Reference
> 8 hr/day	1.40 (0.55, 3.59)	0.23
Average time driving in car/day
≤ 1 hr/day	Reference
> 1 hr/day	0.54 (0.24, 1.21)	0.13
Handwipe TBB
Low (tertile 1)	Reference
Mid (tertile 2)	1.16 (0.42, 3.16)	0.77
High (tertile 3)	1.99 (0.73, 5.41)	0.17
Dust TBB
Low (tertile 1)	Reference
Mid (tertile 2)	1.19 (0.43, 3.35)	0.73
High (tertile 3)	1.55 (0.55, 4.22)	0.40
10^β^ Values represent the multiplicative change in urine concentrations relative to the reference group for categorical variables, or the per-unit change for continuous variables (age).		

**Figure 4:**
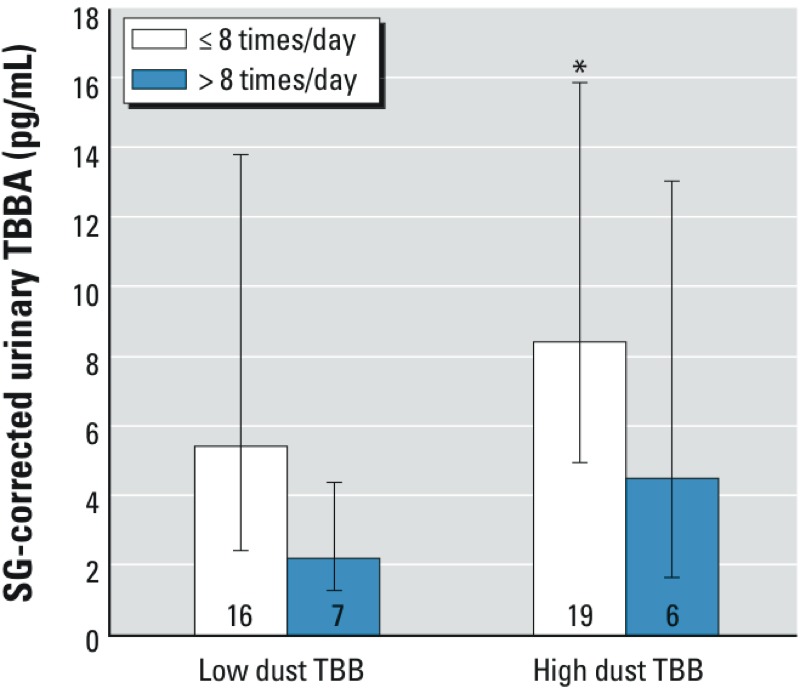
Association [GM (95% CI)] of TBBA measurements in urine with household dust TBB (low, < 275 ng/g; high, > 275 ng/g) and hand-washing frequency (≤ 8 times/day, > 8 times/day). Numbers inside bars indicate the number of samples. *p < 0.05 comparing low-dust/high-wash and high-dust/low-wash groups in regression analyses including an interaction term.

*Dust and urine associations*. The correlation between continuous measures of TBB in dust and TBBA in urine was weakly positive but not statistically significant (*r*_p_ = 0.24; *p* = 0.10; *r*_s_ = 0.20; *p* = 0.16). In bivariate regression models, participants with high levels of TBB in dust samples had urinary TBBA levels 1.55 times those of participants with low dust levels, although results were not statistically significant (tertile 3 vs. tertile 1, 95% CI: 0.55, 4.22; Table 3). Results were similar when dichotomized dust levels were included in the analyses (results not shown). We hypothesized that the relationship between dust TBB and urinary TBBA concentrations may be modified by hand-washing frequency, which bivariate results suggest may be inversely associated with TBBA. We conducted analyses with an interaction term consisting of hand washing and dichotomized house dust. Although the interaction term was not statistically significant (*p* = 0.18), our results indicated that hand washing may modify the association between dust and internal exposure (Figure 4).

*Temporal variation in urinary TBBA*. TBBA levels in repeated urine samples were similar to those from the larger cohort (GM = 5.1 pg/mL; 66.0% detects). Examining temporal variability in SG-corrected TBBA levels using ICCs, we observed moderate reliability over the course of 5 consecutive days (ICC = 0.56; 95% CI: 0.46, 0.66) (Rosner 2000).

## Discussion

Cumulatively, our results indicate that exposures to FM550 are widespread and that the home environment may be an important source of exposure. These data, coupled with the results of recent studies in animals linking exposure to adverse health outcomes (e.g., McGee et al. 2013; Patisaul et al. 2013), prompt the need for additional epidemiologic studies to identify potential impacts of FM550 exposure. Such studies rely on accurate and reliable methods of assessing exposure. On the basis of toxicokinetic investigations using human and animal tissues, we hypothesized that TBBA may be a novel biomarker of FM550 exposure, and we developed a method for measuring urinary TBBA using LC/MS-MS.

We investigated the validity of using TBBA as an indicator of exposure to TBB. In rats, TBBA in urine peaked between exposure and 3 hr after a single dose of FM550, corroborating *in vivo* metabolism studies reporting the rapid metabolism of TBB to TBBA in human and rat tissues (Bearr et al. 2012; Roberts et al. 2012). Rapid formation of TBBA indicates a reduced bioaccumulative potential for TBB and suggests that TBBA may be a useful biomarker of recent FM550 exposure in future studies.

TBBA was frequently detected in urine samples from North Carolina adults (overall detection, 72.4%). Levels of TBBA were highly variable between study participants; one sample had TBBA levels that were 60 times the GM concentration, indicating potential differences in exposure patterns or in TBB metabolism between subjects. We hypothesized that TBB is rapidly metabolized to TBBA; thus, it is likely that the spot urine samples we assessed are reflective of recent exposure (< 1 day previous). Nonetheless, although there was day-to-day variability of TBBA, repeated urine samples collected from the same participants on 5 consecutive days indicate moderate temporal reliability (ICC = 0.56; 95% CI: 0.46, 0.66). Although higher than what might be expected for a rapidly formed metabolite, the estimated ICC for TBBA is similar to what has been reported previously for phthalates (Fromme et al. 2007; Hauser et al. 2004). Our data suggest that the exposure sources (e.g., dust) or the behaviors related to TBB exposure, such as average hand washing frequency, may be stable over time.

*Sources and pathways of FM550 exposure*. Collecting paired house dust, handwip, and urine samples from study participants allowed us to examine associations between sample types and to explore potential pathways of exposure. Our results suggest that higher levels of TBB in house dust may be associated with higher levels of TBBA in urine, thus indicating that the home environment contributes to the overall body burden of TBB. Although associations were generally imprecisely estimated, several lines of evidence point to exposures via the hands as an important pathway of exposure: *a*) higher TBB in dust was associated with a greater mass on participants’ hands, a measure of personal exposure (Table 2); *b*) higher mass of TBB on participants’ hands was also associated with higher levels of TBBA in urine (Table 3); and *c*) frequent hand washing may mitigate the effects of TBB in dust on overall body burden, because participants that reported frequent hand washing had a lower mass of TBB on their hands and had lower levels of TBBA in their urine samples (Figure 1). Although we did not measure internal exposure to TBPH, our results suggest that higher levels of TBPH in household dust may also lead to increased personal exposure. We did not find associations between average hand washing frequency and the levels of TBB or TBPH on participants’ hands; however, handwipe levels may be more closely related to the time since participants last washed their hands rather than their average behavior.

Although our results suggest that TBB on hands may capture individual exposure information and may be an important exposure pathway, in the present study we were unable to determine the mechanism by which TBB gets into the human body. Incidental ingestion via hand-to-mouth contact and dermal absorption are both consistent with our findings and may be similar to an exposure pathway for PBDEs (Johnson et al. 2010; Stapleton et al. 2012; Watkins et al. 2011). However, data suggest that diet can also be a route of exposure for PBDEs (Schecter et al. 2006), which was not investigated in our study. To our knowledge, there are no published assessments of FM550 in food.

*Comparison with previous studies*. We were able to measure detectable levels of TBB and TBPH in the vast majority of household dust samples, suggesting widespread use of FM550 and probable human exposure. Dust TBB and TBPH levels were highly variable, ranging over several orders of magnitude, and were slightly higher than those reported in samples collected in 2006 (Stapleton et al. 2008a) and 2011 (Dodson et al. 2012). Levels of TBB and TBPH in the present study were approximately one order of magnitude lower than the sum of pentaBDE congeners found in household dust samples reported in previous studies [congeners included in the sum of pentaBDEs varied across studies (Dodson et al. 2012; Stapleton et al. 2012; Watkins et al. 2011)]; however, levels in house dust may be expected to increase as older household products containing PBDEs are replaced with newer versions containing FM550, particularly as products age and degrade.

In the present study, we detected TBB and TBPH on the hands of nearly all study participants. On average, the levels of TBB on handwipes were slightly lower than those reported in our previous work investigating the levels of TBB on toddlers’ hands (GM = 31.4 vs. 4.1 ng; Stapleton et al. 2014); differences in behavior or in hand surface area between the two studies may explain differences in the levels of TBB observed. PBDEs have been measured in handwipes in several studies. In contrast to dust, the mass of TBB and TBPH on handwipes in our analyses was similar to results of previous studies reporting the mass of the sum of pentaBDE congeners (Stapleton et al. 2012; Watkins et al. 2011).

Although both TBB and TBPH are components of FM550, it is possible that the TBB and TBPH that we measured are from an alternate source. For example, both compounds are also used in BZ-54, an FR mixture that is also manufactured by Chemtura. However, our previous studies, which consistently found TPP in foam samples containing TBB and TBPH, suggested that FM550 is used more frequently in household foam applications than is BZ-54 (which does not contain TPP) (Stapleton et al. 2009, 2011). TBPH is also used in polyvinyl chloride, neoprene, wire insulation, carpet backing, coated fabrics, wall coverings, and another flame retardant mixture, DP-45 (Covaci et al. 2011). However, the strong correlations between TBB and TBPH in environmental samples suggest a common source. Further data are needed to identify FR mixtures and products containing TBB and THBP.

Our results should be interpreted within the context of several limitations. Our small sample size limited the number of predictive variables that we could include in multivariate regression analyses at the same time and may have limited our power to detect meaningful associations. In addition, paired dust, handwipe, and urine samples were each collected only once. Multiple samples taken over time and in different microenvironments (e.g., workplaces and car) may provide additional insights as to important routes of TBB and TBPH exposure (Watkins et al. 2012). Similarly, household dust samples were collected by participants. Detailed instructions for dust collection were provided; however, variability in the areas sampled, the types of flooring in those areas, and the types of vacuums used may have introduced measurement error into our analyses. Finally, although participants were recruited from the North Carolina general population, the cohort was comprised of a relatively homogeneous group; participants were primarily white and there was little variability in behavioral characteristics. Although this may limit our ability to generalize results to the broader U.S. population, it does not impact the internal validity of our study.

## Conclusions

Cumulatively, our results indicate that the general population is exposed to FM550 in the home environment. These results are of concern given recent toxicological data suggesting that FM550 exposure may have the potential to adversely impact health (e.g., Patisaul et al. 2013). Our results indicate that urinary TBBA provides a useful biomarker of FM550 exposure for future epidemiologic studies. In addition, handwipe levels of TBB were significantly associated with both dust levels and urinary TBBA levels, suggesting that collection and analysis of handwipes may also provide a good measure of exposure.

## Supplemental Material

(463 KB) PDF

## References

[r1] Ali N Dirtu AC Van den Eede N Goosey E Harrad S Neels H 2012 Occurrence of alternative flame retardants in indoor dust from New Zealand: indoor sources and human exposure assessment. Chemosphere 88 1276-1282 22551874 10.1016/j.chemosphere.2012.03.100

[r2] Animal Welfare Act. (2012). 7 USC 2131–2159.. http://www.gpo.gov/fdsys/pkg/USCODE-2012-title7/html/USCODE-2012-title7-chap54.htm.

[r3] Bearr JS, Mitchelmore CL, Roberts SC, Stapleton HM (2012). Species specific differences in the *in vitro* metabolism of the flame retardant mixture, Firemaster® BZ-54.. Aquat Toxicol.

[r4] Boeniger MF, Lowry LK, Rosenberg J (1993). Interpretation of urine results used to assess chemical exposure with emphasis on creatinine adjustments: a review.. Am Ind Hyg Assoc J.

[r5] Churchwell MI, Camacho L, Vanlandingham MN, Twaddle NC, Sepehr E, Delclos KB (2014). Comparison of life-stage-dependent internal dosimetry for bisphenol A, ethinyl estradiol, a reference estrogen, and endogenous estradiol to test an estrogenic model of action in Sprague-Dawley rats.. Toxicol Sci.

[r6] Costa LG, Giordano G, Tagliaferri S, Caglieri A, Mutti A (2008). Polybrominated diphenyl ether (PBDE) flame retardants: environmental contamination, human body burden and potential adverse health effects.. Acta Biomed.

[r7] Covaci A, Harrad S, Abdallah MA, Ali N, Law RJ, Herzke D (2011). Novel brominated flame retardants: a review of their analysis, environmental fate and behaviour.. Environ Int.

[r8] de Wit CA (2002). An overview of brominated flame retardants in the environment.. Chemosphere.

[r9] Dodson RE, Perovich LJ, Covaci A, Van den Eede N, Ionas AC, Dirtu AC (2012). After the PBDE phase-out: a broad suite of flame retardants in repeat house dust samples from California.. Environ Sci Technol.

[r10] Fromme H, Bolte G, Koch HM, Angerer J, Boehmer S, Drexler H (2007). Occurrence and daily variation of phthalate metabolites in the urine of an adult population.. Int J Hyg Environ Health.

[r11] Hauser R Meeker JD Park S Silva MJ Calafat AM 2004 Temporal variability of urinary phthalate metabolite levels in men of reproductive age. Environ Health Perspect 112 1734 1740; 10.1289/ehp.7212 15579421 PMC1253667

[r12] James GD, Sealey JE, Alderman M, Ljungman S, Mueller FB, Pecker MS (1988). A longitudinal study of urinary creatinine and creatinine clearance in normal subjects. Race, sex, and age differences.. Am J Hypertens.

[r13] Johnson PI, Stapleton HM, Sjodin A, Meeker JD (2010). Relationships between polybrominated diphenyl ether concentrations in house dust and serum.. Environ Sci Technol.

[r14] La Guardia MJ, Hale RC, Harvey E, Chen D (2010). Flame-retardants and other organohalogens detected in sewage sludge by electron capture negative ion mass spectrometry.. Environ Sci Technol.

[r15] Ma Y, Venier M, Hites RA (2012). 2-Ethylhexyl tetrabromobenzoate and bis(2-ethylhexyl) tetrabromophthalate flame retardants in the Great Lakes atmosphere.. Environ Sci Technol.

[r16] McGee SP, Konstantinov A, Stapleton HM, Volz DC (2013). Aryl phosphate esters within a major pentaBDE replacement product induce cardiotoxicity in developing zebrafish embryos: potential role of the aryl hydrocarbon receptor.. Toxicol Sci.

[r17] National Research Council. (2011). Guide for the Care and Use of Laboratory Animals. 8th ed. Washington, DC:National Academies Press.. http://www.nap.edu/catalog.php?record_id=12910.

[r18] Patisaul HB, Roberts SC, Mabrey N, McCaffrey KA, Gear RB, Braun J (2013). Accumulation and endocrine disrupting effects of the flame retardant mixture Firemaster® 550 in rats: an exploratory assessment.. J Biochem Mol Toxicol.

[r19] Patisaul HB Sullivan AW Radford ME Walker DM Adewale HB Winnik B 2012 Anxiogenic effects of developmental bisphenol A exposure are associated with gene expression changes in the juvenile rat amygdala and mitigated by soy. PLoS One 7 9 e43890; 10.1371/journal.pone.0043890 22957036 PMC3434201

[r20] Roberts SC, Macaulay LJ, Stapleton HM (2012). *In vitro* metabolism of the brominated flame retardants 2-ethylhexyl-2,3,4,5-tetrabromobenzoate (TBB) and bis(2-ethylhexyl) 2,3,4,5-tetrabromophthalate (TBPH) in human and rat tissues.. Chem Res Toxicol.

[r21] Rosner B. (2000). Fundamentals of Biostatistics. 5th ed..

[r22] Schecter A Päpke O Harris TR Tung KC Musumba A Olson J 2006 Polybrominated diphenyl ether (PBDE) levels in an expanded market basket survey of U.S. Food and estimated PBDE dietary intake by age and sex. Environ Health Perspect 114 1515 1520; 10.1289/ehp.9121 17035135 PMC1626425

[r23] Shrout PE, Fleiss JL (1979). Intraclass correlations: uses in assessing rater reliability.. Psychol Bull.

[r24] Stapleton HM, Allen JG, Kelly SM, Konstantinov A, Klosterhaus S, Watkins D (2008a). Alternate and new brominated flame retardants detected in U.S. house dust.. Environ Sci Technol.

[r25] Stapleton HM Eagle S Sjödin A Webster TF 2012 Serum PBDEs in a North Carolina toddler cohort: associations with handwipes, house dust, and socioeconomic variables. Environ Health Perspect 120 1049 1054; 10.1289/ehp.1104802 22763040 PMC3404669

[r26] Stapleton HM, Kelly SM, Allen JG, McClean MD, Webster TF (2008b). Measurement of polybrominated diphenyl ethers on hand wipes: estimating exposure from hand-to-mouth contact.. Environ Sci Technol.

[r27] Stapleton HM, Klosterhaus S, Eagle S, Fuh J, Meeker JD, Blum A (2009). Detection of organophosphate flame retardants in furniture foam and U.S. house dust.. Environ Sci Technol.

[r28] Stapleton HM, Klosterhaus S, Keller A, Ferguson PL, van Bergen S, Cooper E (2011). Identification of flame retardants in polyurethane foam collected from baby products.. Environ Sci Technol.

[r29] Stapleton HM Misenheimer JC Hoffman K Webster TF 2014 Flame retardant associations between children’s handwipes and house dust. Chemosphere; 10.1016/j.chemosphere.2013.12.100 PMC4116470 24485814

[r30] Taylor JA Vom Saal FS Welshons WV Drury B Rottinghaus G Hunt PA 2011 Similarity of bisphenol A pharmacokinetics in rhesus monkeys and mice: relevance for human exposure. Environ Health Perspect 119 422 430; 10.1289/ehp.1002514 20855240 PMC3080921

[r31] U.S. Environmental Protection Agency. (2009). Polybrominated Diphenyl Ethers (PBDEs) Action Plan.. http://epa.gov/oppt/existingchemicals/pubs/actionplans/pbdes_ap_2009_1230_final.pdf.

[r32] Van den Eede N, Dirtu AC, Ali N, Neels H, Covaci A (2012). Multi-residue method for the determination of brominated and organophosphate flame retardants in indoor dust.. Talanta.

[r33] van der Veen I, de Boer J (2012). Phosphorus flame retardants: properties, production, environmental occurrence, toxicity and analysis.. Chemosphere.

[r34] Watkins DJ McClean MD Fraser AJ Weinberg J Stapleton HM Sjödin A 2011 Exposure to PBDEs in the office environment: evaluating the relationships between dust, handwipes, and serum. Environ Health Perspect 119 1247 1252; 10.1289/ehp.1003271 21715243 PMC3230398

[r35] Watkins DJ, McClean MD, Fraser AJ, Weinberg J, Stapleton HM, Sjödin A (2012). Impact of dust from multiple microenvironments and diet on pentaBDE body burden.. Environ Sci Technol.

