# Supplementary material for: Urinary Tetrabromobenzoic Acid (TBBA) as a Biomarker of Exposure to the Flame Retardant Mixture Firemaster® 550
Source: Environ Health Perspect. 2014 May 13;122(9):963–9. doi: 10.1289/ehp.1308028 (PMC4154220; doi:10.1289/ehp.1308028)
Supplement: (463 KB) PDF [file ehp.1308028.s001.pdf]

Supplemental Material

Urinary Tetrabromobenzoic Acid (TBBA) as a Biomarker of Exposure to the Flame Retardant Mixture Firemaster® 550

Kate Hoffman, Mingliang Fang, Brian Horman, Heather B. Patisaul, Stavros Garantziotis,

Linda S. Birnbaum, and Heather M. Stapleton

Table of Contents **Page**

Dust and handwipe analyses 2

Method development of TBBA analysis in human urine 3

Figure S1. Response of surrogate standard TIBA (5 ng) in 10 mL urine extracts performed by Agilent SampliQ OPT (n = 7) and liquid-liquid extraction (n = 10) with hexane relative to 5 ng TIBA standard in pure solvent with the same volume. 4

Figure S2. Mean cumulative mass of TBBA in urine samples (ng) collected from 6 treated and 6 control adult rats. 5

Figure S3. Instructions for the collection of a house dust sample. 6-7

References 8

Dust and handwipe analyses

Handwipe and dust samples were extracted in the laboratory and analyzed for TBB and TBPH. Each handwipe sample was extracted using a Soxhlet apparatus. Prior to Soxhlet extraction, handwipes were spiked with a monofluorinated tetrabrominated diphenyl ether (F-BDE-69; 50 ng) as an internal standard. To serve as laboratory blanks, new sterile gauze pads were taken through the same procedure and run next to handwipe samples. Samples were Soxhlet extracted with 50:50 dichloromethane (DCM):hexane, after which each extract was concentrated using an automated nitrogen evaporation system (Turbo Vap II, Zymark Inc.) and transferred to a 4.0 mL amber vial, stored in a -20ºC freezer. Extracts were then cleaned using Florisil solid-phase extraction (Supelclean ENVI-Florisil, 6mL, 500mg bed weight, Supelco), eluting with 10mL hexane based on the method developed by Van den Eede et al (Van den Eede et al. 2012). Each fraction was then concentrated to approximately 1 mL using a nitrogen evaporation system and transferred to an autosampler vial (ASV) for GC/MS analysis. Dust samples (~100 mg) were first spiked with FBDE-69 as an internal standard and extracted with 10 mL of 50:50 DCM:hexane using sonication. This process was repeated three times and the combined extract (~30 mL) was concentrated using an automated nitrogen evaporation system and transferred to a 4.0 mL amber vial, stored in a -20ºC freezer. The dust extracts were cleaned using the same method as described for the handwipe samples above. To measure recovery of F-BDE-69 (Chiron, Norway) extracts were spiked with ^{13}C-CDE 141 (Wellington Laboratories). Analysis of laboratory blanks (n=5) and an indoor dust Standard Reference Materials (SRM 2585, NIST, Gaithersburg, MD) were employed for quality assurance and quality control.

Method development of TBBA analysis in human urine

In our previous *in vitro* study on TBBA (Roberts et al. 2012), 6 mL Agilent SampliQ OPT SPE columns were used to concentrate and clean microsomal samples. Therefore, SPE method was first tested for efficiency in terms of extracting TBBA in urine samples. However, a significant matrix effect, i.e., ion suppression of TBBA and TIBA was observed, which may be due to the large volume of urine (~ 10 mL) used in each sample. As shown in Figure S1, the response of TIBA in urine samples was approximately 20% of the TIBA standard in pure solvent with the same volume. A similar matrix effect was also observed with other reverse-phase SPE columns (e.g., StrataX-AW Phenomenex, data not shown). Liquid-liquid extraction has been suggested as an effective method to reduce the matrix effect, yielding rather clean extracts. In this study, hexane, dichloromethane, ethyl acetate, and MTBE were tested individually to extract TBBA from urine. Sulfuric acid was added to neutralize TBBA and facilitate the extraction, which might also help hydrolyze possible TBBA conjugates, if they existed, and minimize the matrix. In a phosphate buffer (PBS, pH: 7.4, 0.1 M) spiked with 1, 5, and 10 ng TBBA, > 80% (n = 3 for each) of the spiked TBBA was recovered. Though other solvents might have better extraction efficacy, hexane extracts showed the least matrix and was used as the extraction solvent. The matrix effect of TBBA in urine was further investigated using a matrix spike approach. A set of urine samples from one pooled urine (in which TBBA was undetected), were spiked with three different levels of TBBA (i.e., 1, 5, and 10 ng, in triplicate) and extracted with hexane using the above-mentioned liquid-liquid extraction technique. The average recovery of the TBBA was 103%, 98% and 102% of the spiked amount, respectively. In another 10 different urine samples, ion suppression was significantly reduced and the response of TIBA in those samples was ~ 80% of the pure standard (Figure S1), suggesting the current liquid-liquid extraction method could

effectively eliminate a majority of the matrix. Since a labeled recovery standard was not available in this study, recovery of TBBA in urine was further examined by spiking 1 ng of TBBA into 10 urine samples in which no TBBA was detected. Recovery of TBBA was estimated by adding TIBA post extraction and cleanup instead of as the surrogate standard. The calculated recovery averaged ± SD of 79 ± 9 % (n = 10).

PBS blanks were run throughout the process as controls. TBBA was not detected in either field or laboratory blanks. Therefore, the method detection limit (MDL) was estimated by measuring the instrumental detection limit (IDL), which was calculated by using a signal-to-noise (S/N) ratio of 3 and a volume of 10 mL. In this study, the MDL of TBBA in urine samples was calculated to be 5 pg/mL urine.

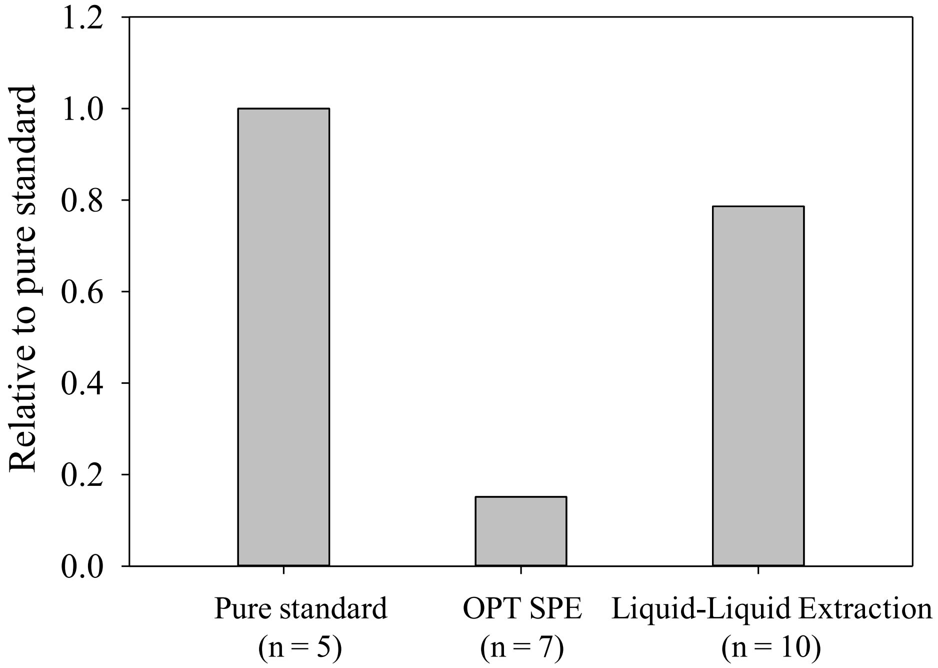

Figure S1. Response of surrogate standard TIBA (5 ng) in 10 mL urine extracts performed by Agilent SampliQ OPT (n = 7) and liquid-liquid extraction (n = 10) with hexane relative to 5 ng TIBA standard in pure solvent with the same volume.

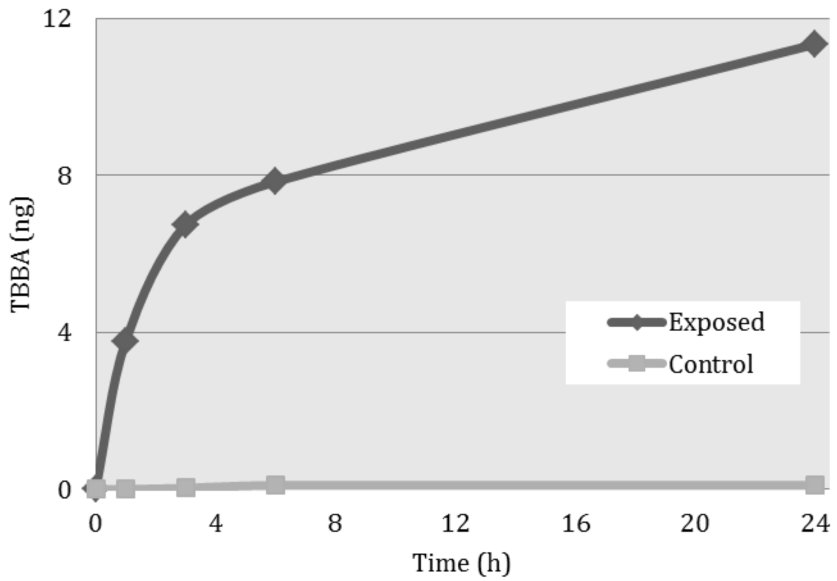

Figure S2. Mean cumulative mass of TBBA in urine samples (ng) collected from 6 treated and 6 control adult rats. Exposed animals were administered a one-time dose of 1mg Firemaster 550® (via treat pellet) at time zero. TBBA concentrations were measure at 1, 3, 6, and 24 hours in urine sample from exposed animals and controls.

Dust Collector Instruction Card

Read Steps 1 – 8 before starting

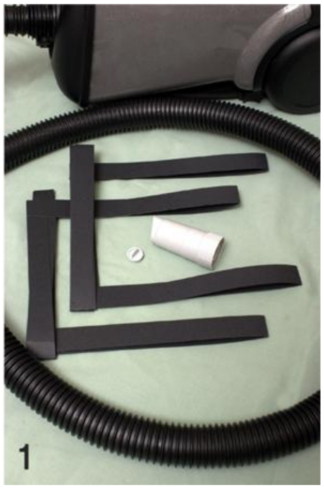

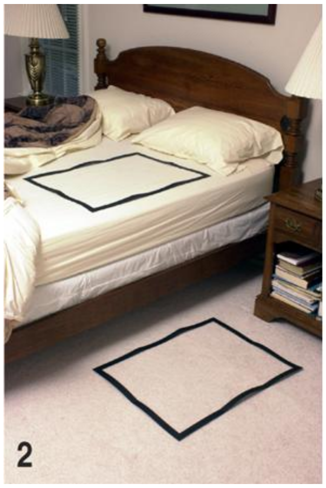

1. **Bring to your livingroom:**
   The Dust Collector and its cap and a watch or clock for timing

2. **Place the Dust Collector on the end of your vacuum hose (it may fit loosely until the vacuum is turned on).**

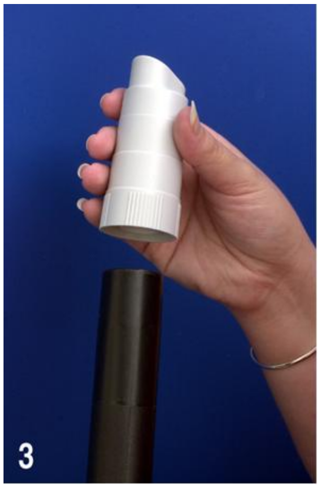

3. **Prepare your watch or clock for timing.**

   Vacuum the area within one square for exactly 2 minutes.

   Without turning the vacuum off, continue to Step 5.

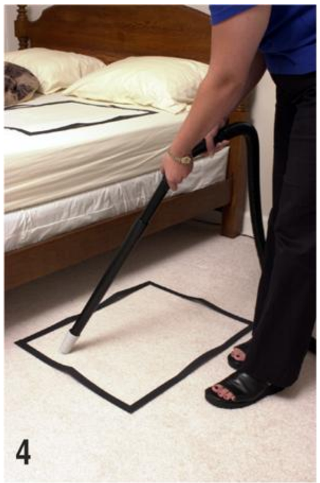

4. **Vacuum the area within the other square for exactly 2 minutes**

Continued on the other side

Dust Collector Instruction Card, continued

5. **While holding the Collector up, turn the vacuum off.**

   Push the cap firmly onto the top of the Collector.

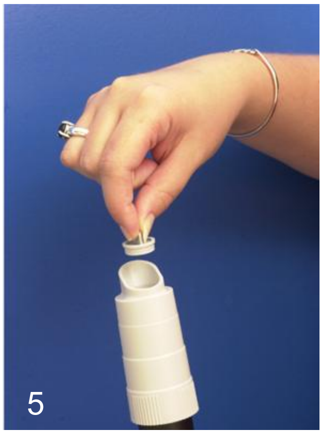

6. **Remove the Collector from the hose and place it back in the Ziplock bag and close.**

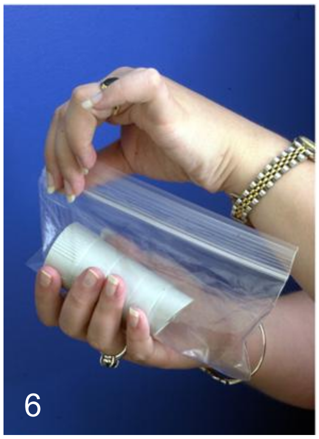

7. **Place the following items in the return mailing envelope:**
   - **The Ziplock bag containing the Dust Collector**

8. **Place the return envelope in the U.S. mail within 12 hours**

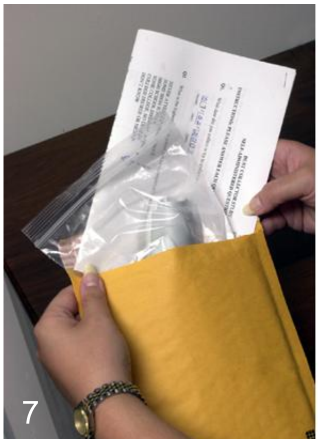

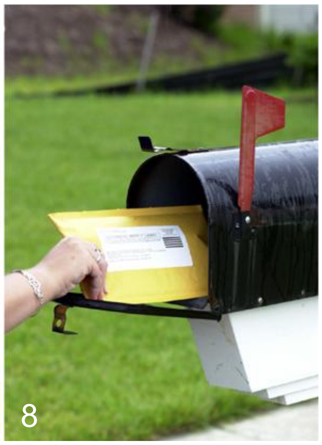

Figure S3. Instructions for the collection of a house dust sample.

References

Roberts SC, Macaulay LJ, Stapleton HM. 2012. In vitro metabolism of the brominated flame retardants 2-ethylhexyl-2,3,4,5-tetrabromobenzoate (TBB) and bis(2-ethylhexyl) 2,3,4,5-tetrabromophthalate (TBPH) in human and rat tissues. Chemical research in toxicology 25:1435-1441.

Van den Eede N, Dirtu AC, Ali N, Neels H, Covaci A. 2012. Multi-residue method for the determination of brominated and organophosphate flame retardants in indoor dust. Talanta 89:292-300.
